# Unique Running Pattern and Mucosal Morphology Found in the Colon of Cotton Rats

**DOI:** 10.3389/fphys.2020.587214

**Published:** 2020-10-26

**Authors:** Tsolmon Chuluunbaatar, Osamu Ichii, Teppei Nakamura, Takao Irie, Takashi Namba, Md Rashedul Islam, Yuki Otani, Md Abdul Masum, Yuko Okamatsu-Ogura, Yaser Hosny Ali Elewa, Yasuhiro Kon

**Affiliations:** ^1^Laboratory of Anatomy, Department of Basic Veterinary Sciences, Faculty of Veterinary Medicine, Hokkaido University, Sapporo, Japan; ^2^Department of Basic Science of Veterinary Medicine, School of Veterinary Medicine, Mongolian University of Life Science, Ulaanbaatar, Mongolia; ^3^Laboratory of Agrobiomedical Science, Faculty of Agriculture, Hokkaido University, Sapporo, Japan; ^4^Department of Biological Safety Research, Chitose Laboratory, Japan Food Research Laboratories, Chitose, Japan; ^5^Medical Zoology Group, Department of Infectious Diseases, Hokkaido Institute of Public Health, Sapporo, Japan; ^6^Laboratory of Veterinary Parasitic Diseases, Department of Veterinary Sciences, Faculty of Agriculture, University of Miyazaki, Miyazaki, Japan; ^7^Department of Surgery and Theriogenology, Faculty of Animal Science and Veterinary Medicine, Sher-e-Bangla Agricultural University, Dhaka, Bangladesh; ^8^Department of Anatomy, Histology and Physiology, Faculty of Animal Science and Veterinary Medicine, Sher-e-Bangla Agricultural University, Dhaka, Bangladesh; ^9^Laboratory of Biochemistry, Department of Basic Veterinary Sciences, Faculty of Veterinary Medicine, Hokkaido University, Sapporo, Japan; ^10^Department of Histology, Faculty of Veterinary Medicine, Zagazig University, Zagazig, Egypt

**Keywords:** cotton rat, morphology, large intestine, colon, longitudinal fold, sex difference

## Abstract

Cotton rats are one of the experimental rodents used for testing different infectious and non-infectious diseases, including gastrointestinal tract pathology. However, their intestinal morphological characteristics are still poorly understood. Here, we clarified the anatomical and histological characteristics of the cecum and ascending colon (AC) of young (1–3-month old), adult (4–6-month old), and old (10–12-month old) cotton rats. The large intestine (LI) in cotton rats is composed of the cecum, AC, transverse and descending colons, and rectum, and is similar to that of other mammals. The AC begins with a double or triple spiral loop-like flexure (SLLF) and ends with a coupled horseshoe-like flexure (HSLF). A single longitudinal mucosal fold (SLMF) was found at the beginning of the AC along the mesentery line and developed with age. Furthermore, the SLMF contained several lymphatic nodules (LNs), indicating their role in digestive and immunological functions. Small and large protuberant LNs were found in the cecum and SLLF, respectively, whereas thin and flat LNs were observed in the HSLF and transverse colon, respectively. Regarding sex-related differences, adult females had a significantly longer AC with a higher number of SLLFs compared to males. The SLMF length and LN number were also longer and higher, respectively, in adult females compared to adult males. These are crucial findings, indicating the presence of sex-related differences in the morphology of the LI in cotton rats, and ours is the first study to discover a sex difference in the mammalian LI lining. Our study clarified the unique morphology of the LI in cotton rats, which could serve as the principal model for elucidating species-specific digestive tract functions and gastrointestinal disorders.

## Introduction

The overall gastrointestinal tract (GIT) morphology is considerably affected by adaptation, feeding behavior, food consumption regularity, food stocking, and individual body size and shape (Kararli, 1995). The GIT morphology of herbivorous animals is organized to supply fermentation areas for the digestion of plant fibers (Stevens and Hume, 1998). Smaller animals have relatively higher energy requirements than larger animals (Kleiber, 1975). Herbivorous rodents show great differences around the cecocolic area, with the fermentation of fibers occurring primarily in the cecum and sometimes in the colon (Hume et al., 1993; Langer, 2002; Sakaguchi, 2003). Large fiber particles that are hard to digest are directed toward the ascending colon (AC), and are passed quickly. Liquid and small fiber particles, which are easier to digest, are directed toward the cecum, remaining there for a long time (Hume et al., 1993).

There are large species-specific differences in rodent GIT morphology. Strict herbivore rodents, such as Brandt’s vole (*Microtus brandti*), that eat herbs, leaves, roots, grasses, and stems, have a comparatively longer and larger GIT than that of the desert hamster (*Phodopus roborovskii*), which mainly eats seeds and invertebrates (Wang et al., 2003). As for the AC, the cecum of *Meriones* and *Gerbillus* species is completely isolated due to the development of a straight path from the ileum to the colon (Naumova and Kucheruk, 1996; Zharova et al., 2010; Naumova et al., 2019). Sundevall’s jird (*Meriones crassus*) and the fat sand rat (*Psammomys obesus) have a* prolonged cecum with curved apex, ileum, and colon located close to each other, forming a unified opening, and the V-shaped colon forming a loop. African muroid rodent species (*Rhabdomys dilectus, R*. *pumilio, Aethomys chrysophilus*, and *Lemniscomys rosalia*) show short, solitary colonic loops in the AC that folded in on itself (Henke et al., 2018).

In addition to these external features, the internal structure of the GIT also differs among species. The GIT mucosa possesses many folds, grooves, and furrows, which have different structures and functions depending on the species to which they belong. Characteristically, the rabbit has spiral mucosal folds in the cecum up to the vermiform appendix (Stan et al., 2014), their function being related to channeling digesta and increasing absorption area. Rodents such as nutrias (*Myocastor coypus*) and African mole rats (Bathyergidae) possess colonic furrows, which increase bacterial density in the digesta in order to facilitate coprophagy (Takahashi and Sakaguchi, 2000; Kotze et al., 2006).

The size and location of gut-associated lymphoid tissue differ among species. While rats and mice possess uniform-sized follicles, pigs, dogs, and ruminants have two different types of follicles, which are distinct and appear as long, continuous patches in the GIT. Thus, the jejunum and upper ileum generally possess distinct patches, while the terminal ileum includes long continuous patches (Haley, 2017).

Our previous studies clarified several unique morphological phenotypes in cotton rats, such as the existence of pharyngeal pouch remnant, visceral fat tissue inflammation, abnormal adipose tissue accumulation in the pancreas without obesity, development of chronic kidney disease, and pyometra (Ichii et al., 2016, 2018, 2020; Nakamura et al., 2018, 2019). *Sigmodon hispidus* is most commonly used as an experimental animal among the seven species of cotton rats (Faith et al., 1997). Cotton rats were first used in polio research in the 1930s, and are susceptible to various diseases caused by viruses, bacteria, protozoa, and metazoans (Armstrong, 1939).

Thus, there are large species-specific differences in GIT morphology, particularly relating to the AC and its function. The morphological characteristics of cotton rat GITs remain unclear; nevertheless, cotton rats are used for GIT models for diseases such as multi-step carcinogenesis (Kawase and Ishikura, 1995). Therefore, this study aimed to identify species-specific features of the GIT and to discuss its physiological function and pathogenesis. Importantly, in this study, we also found sex-related differences in AC morphology in cotton rats. Our data provide basic biological findings as well as crucial information for understanding sex-related gastrointestinal diseases, such as irritable and inflammatory bowel disease.

## Materials and Methods

### Animal and Sample Preparation

Animal experiments were performed according to the guidelines of the Hokkaido Institute of Public Health (Sapporo, Japan; Approval No.: K27-03). Male and female cotton rats (1–12 months old; HIS/Hiph strain) were maintained under conventional conditions at the Hokkaido Institute of Public Health and continuously inbred. A previous study showed four distinct life stages in cotton rats: 1–75 days; juveniles, 76–200 days; young adults, 201–300 days; adults, and 300 days onward; old adults (Kawase and Ishikura, 1995; Faith et al., 1997). In the present study, cotton rats (*n* = 65) were divided into six groups based on age and sex: young (1–3 months old) males (*n* = 21) and females (*n* = 12), adult (4–6 months old) males (*n* = 11) and females (*n* = 8), and old (10–12 months old) males (*n* = 6) and females (*n* = 7).

For supplemental experiments, we compared a part of the large intestine (LI) morphology among cotton rats, mice (male, C57BL/6N, 2 months old, *n* = 4), and hamsters (male, SLC: Syrian, 6 months old, *n* = 4). The mice and hamsters were purchased from Japan SLC (Hamamatsu, Japan). These animal experiments were approved by the Institutional Animal Care and Use Committee of the Graduate School of Veterinary Medicine, Hokkaido University (approval numbers 18-0052 and 20-0012). Investigators adhered to the Guide for the Care and Use of Laboratory Animals of Hokkaido University, Faculty of Veterinary Medicine.

Cotton rats were placed under deep anesthesia using isoflurane and euthanized by cutting the abdominal aorta. Other animals were euthanized using CO_2_ inhalation. The whole organs of cotton rats and the LI of the other animals were fixed using 10% neutral buffered formalin or Bouin’s solution, and then the GITs of the cotton rats were collected. Body weight was measured. The position of the internal organs in these specimens were photographed and recorded before removing the GITs.

### Morphological Analyses

The lengths of the cecum and AC (spiral loop like flexure, SLLF; horseshoe-like flexure, HSLF) of the LI were measured on the antimesenteric border using a pliable, non-stretchable cord. The intestinal walls were cut open longitudinally along opposite sides of the mesenteric border and gently rinsed out. The length of the single longitudinal mucosal fold (SLMF) found in the LI was measured using a pliable, non-stretchable cord. The diameter of the lymphatic nodule (LN) and height of the SLMF were measured by using an LCD displayed digital caliper having an accuracy of 0.001 mm, (AD-5763-150; A&D Company, Tokyo, Japan) then LN area was calculated. The number of LNs found in the cecum and AC were identified based on their gross anatomy. Photographs were taken using a DMC-FX3799 device (Panasonic; Osaka, Japan).

### Histological Analysis

To evaluate the histology of the LI, each part of the LI was dehydrated and embedded in paraffin. Then, 3-μm paraffin sections were prepared and stained with hematoxylin and eosin. Histological digital images were captured using a BZ-X710 device (Keyence; Osaka, Japan).

### Statistical Analyses

Results are expressed as mean ± standard error. The Mann-Whitney *U* test was used to compare the data between male and female cotton rats (*P* < 0.05). The Kruskal-Wallis test was used to compare data between the age groups, and multiple comparisons were performed using Scheffé’s method when a significant difference was noted (*P* < 0.05).

## Results

### Gross Anatomical Features of the LI in Cotton Rats

**FIGURE 1:**
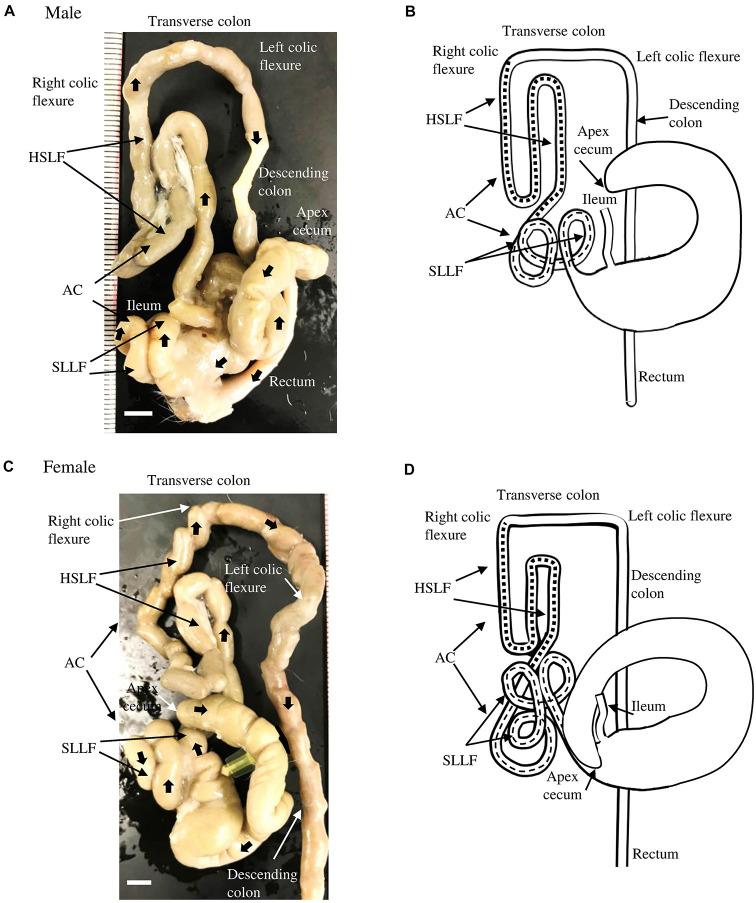
Gross anatomical features of the LI in cotton rats. **(A,B)** Male. Panel **(A)** shows a picture from the ventral view at 6 months old. Panel **(B)** summarizes the morphology and running pattern of the LI from the ventral view. **(C,D)** Female. Panel **(C)** shows a picture from the ventral view at 6 months old. Panel **(D)** summarizes the morphology and running pattern of the LI from the ventral view. Thick arrows represent the direction of intestines toward the rectum **(A,C)**. Flat dashed lines and square dashed lines represent a spiral loop-like flexure (SLLF) and horseshoe-like flexure (HSLF), respectively. LI, large intestine, AC, ascending colon. Ruler = 1 mm.

Figure 1A shows the running pattern of the LI of adult male cotton rats (6 months old) from the ventral view. The ileum was connected to the LI via the ileal orifice, and the left and right sides of the LI from the ileal opening were the cecum and AC, respectively (Figure 1A). The cecal base was mostly located in the middle of the central part of the abdomen toward the left half, and its flexure patterns differed among individual animals. Generally, the well-developed, sacculated, long cecum began by twisting counterclockwise and showed various pattern shapes such as semilunar, sigmoidal, curved, comma, or round. It then became localized to the caudal part of the abdominal cavity, and finished with a spacious apex. A vermiform appendix was not observed at the end of the cecum and its wall was thinner (data not shown) compared to other parts of the LI.

Opposite the ileal opening, the beginning of the AC showed a large diameter, similar to the cecum, and ran to the right half of the abdomen. It then suddenly decreased in diameter just before running to the cranial side (Figure 1A). The subsequent AC showed unique and complex flexures. Briefly, on the right half of the abdomen, several spiral loops were observed, which were tightly connected to each other by connective tissue and mesentery (Figure 1A). These SLLFs began on the right caudoventral side of the abdomen (just after the AC decreased in diameter) and ran to the caudodorsal side, making double or triple loops. Then, the AC ran slightly to the caudal ventral side, and diagonally in the cranial direction. Subsequently, the AC continued in to a dorsal-lying HSLF toward the right half of the abdomen by running in the cranial, caudal, and again cranial directions. At the right dorsal cranial side of the abdomen, the HSLF was connected to the right colic flexure. A relatively short transverse colon then crossed cranial dorsal to the root of the mesentery, connected to the left colic flexure, and continued caudally to the descending colon, which has a higher fat content than the jejunum. By entering the pelvis, the descending colon became the rectum (Figure 1A). The running pattern of the LI found in male cotton rats is summarized in Figure 1B.

The morphology of the LI in female cotton rats was similar to that in males (Figure 1C), however, the number of SLLFs was significantly higher in females than in males. The LI of male cotton rats mostly contained two loops; however, in female rats, there were three. The running pattern of the LI in female cotton rats is summarized in Figure 1D.

For the LI of the other rodents, the SLLF was identified in hamsters and found to be similar to that in cotton rats, but not in mice (Supplementary Figures S1A–C). The HSLF was identified in both mice and hamsters, however, their lengths were significantly shorter and longer, respectively, than those of the cotton rats, and the latter showed a quadruple pattern in the HSLF. Hamsters and mice did not show sex differences in LI morphology (data not shown).

### Morphometry of the Cecum and AC in Cotton Rats

**FIGURE 2:**
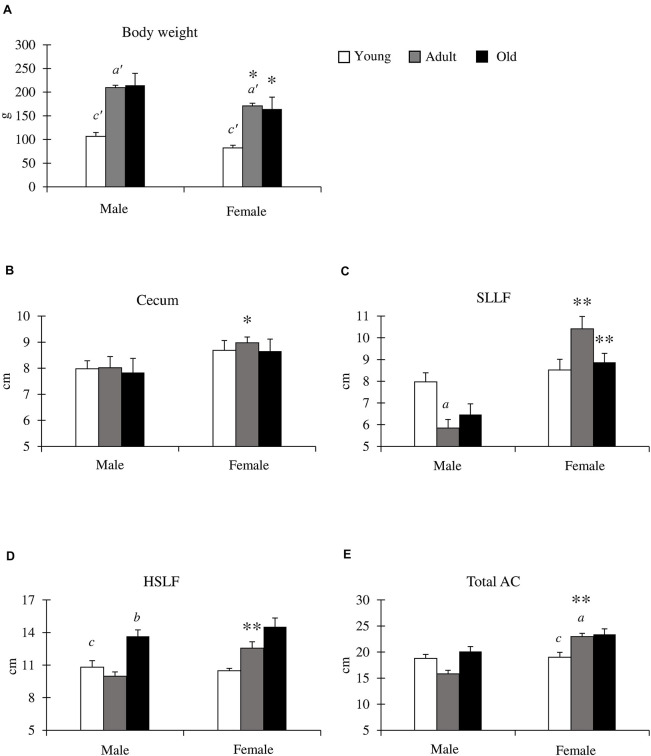
Body weight, and cecum and ascending colon (AC) lengths in cotton rats. **(A)** Body weight. Significant differences between the young and adult groups are indicated by *a’*. Significant differences between the young and old groups are indicated by *c’.* (*a’, c’ P* < 0.01; Kruskal-Wallis test followed by Scheffé’s method). **(B–E)** Lengths of cecum **(B)**, spiral loop-like flexure (SLLF) **(C)**, horseshoe-like flexure (HSLF) **(D)**, and total AC **(E)**. Values = mean ± standard error. *n* = 21 (male, young age group), *n* = 12 (female, young age group), *n* = 11 (male, adult age group), *n* = 8 (female, adult age group), *n* = 6 (male, old age group), and *n* = 7 (female, old age group). Significant sex-related differences in the same age group are indicated by ^∗^*P* < 0.05 or ^∗∗^*P* < 0.01 (Mann-Whitney *U* test). Significant differences between the young and adult groups are indicated by *a*. Significant differences between the adult and old groups are indicated by *b.* Significant differences between the old and young groups are indicated by *c* (*a*, *b, c P* < 0.05; Kruskal-Wallis test followed by Scheffé’s method).

We compared the body weight and cecum, SLLF, and HSLF lengths between sexes and among the young (1–3 months old), adult (4–6 months old), and old (10–12 months old) groups (Figure 2). Body weight was significantly increased from the adult period onward in both sexes, and the body weight of males was significantly higher than that of females in the adult and old groups (Figure 2A). The length of the measured LI was not related to body weight. For the cecum lengths, there were no significant differences among the groups for both sexes, however, the female ceca were longer than those of the males, and a significant sex-related difference was observed in the adult groups (Figure 2B). In contrast, the SLLF length differed between the sexes and groups (Figure 2C). The SLLF was longer in the young group than in the other groups, and significant differences were observed between the young and adult male groups. The lengths of the SLLF were significantly longer in female rats than in male rats in the adult and old groups without any age-related significant differences. The HSLF length was the highest in the old group for both sexes, and males showed significant differences between the old group and other groups in HSLF length. The adult group had significantly longer HSLFs in females than in males (Figure 2D). Figure 2E shows the total AC length. There were no age-related AC differences in males, however, females had significantly longer AC lengths in the adult and old groups compared to those in the young group. Additionally, the adult group had a significantly longer AC length in females than in males.

We calculated the length of the small intestine and the LI ratio in mice, cotton rats, and hamsters (Supplementary Figures S2A–D). The small intestine and LI ratio of the cotton rats were significantly higher than those of hamsters, and significantly lower than those of mice (Supplementary Figure S2D).

### Detailed Morphology of the SLLF in Cotton Rats

**FIGURE 3:**
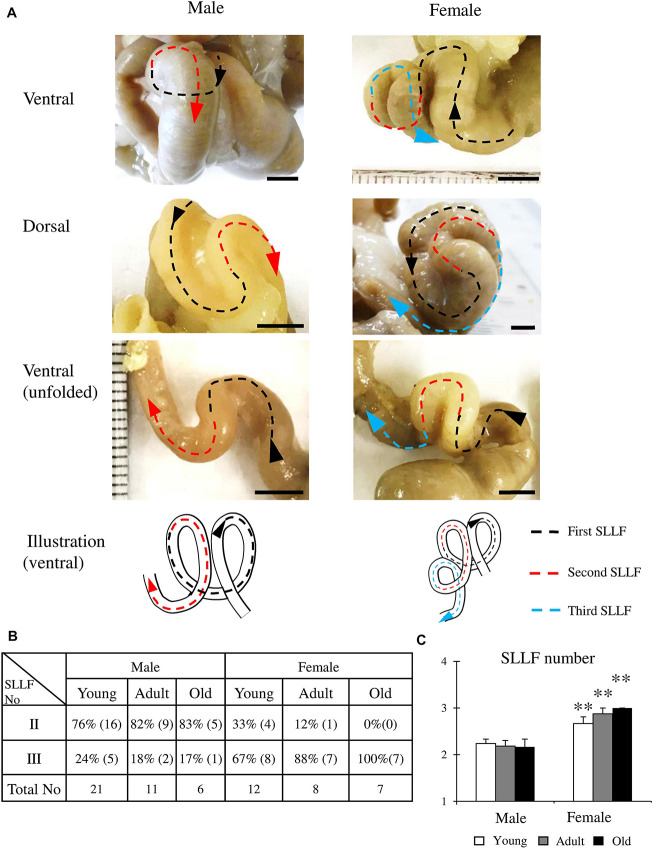
Morphological features of the spiral loop-like flexure (SLLF) in the ascending colon (AC) of cotton rats. **(A)** Sex-related differences of the SLLF from the ventral and dorsal views. Black, red, and blue arrows indicate the first and second and their flexures of SLLF with direction toward the rectum. **(B,C)** Number of SLLFs regarding appearance **(B)** and mean values **(C)**. Values = percentage **(B)** or mean ± standard error **(C)**. *n* = 21 (male, young age group), *n* = 12 (female, young age group), *n* = 11 (male, adult age group), *n* = 8 (female, adult age group), *n* = 6 (male, old age group), and *n* = 7 (female, old age group). Significant sex-related differences in the same age group are indicated by ^∗∗^*P* < 0.01; Mann-Whitney *U* test.

Next, we focused on the morphology of the SLLF, which had a curved, loop-like structure. Figure 3 shows the representative adult (6 months old) SLLF of both sexes from the ventral and dorsal aspects. The males and females usually had two and three flexures, respectively, and the SLLF of the females was longer and more complicated than that of males (Figure 3A). Figure 3B summarizes the number of flexures in the SLLF of both sexes. Almost all males had two flexures (76–83%), whereas the adult and old female groups had three flexures (88 and 100%, respectively), with only some of the young female group having two flexures (33%). From the comparison of the average number of flexures in the SLLF, females showed significantly higher values than males in all examined groups (Figure 3C).

### Inner Features of the AC in Cotton Rats

**FIGURE 4:**
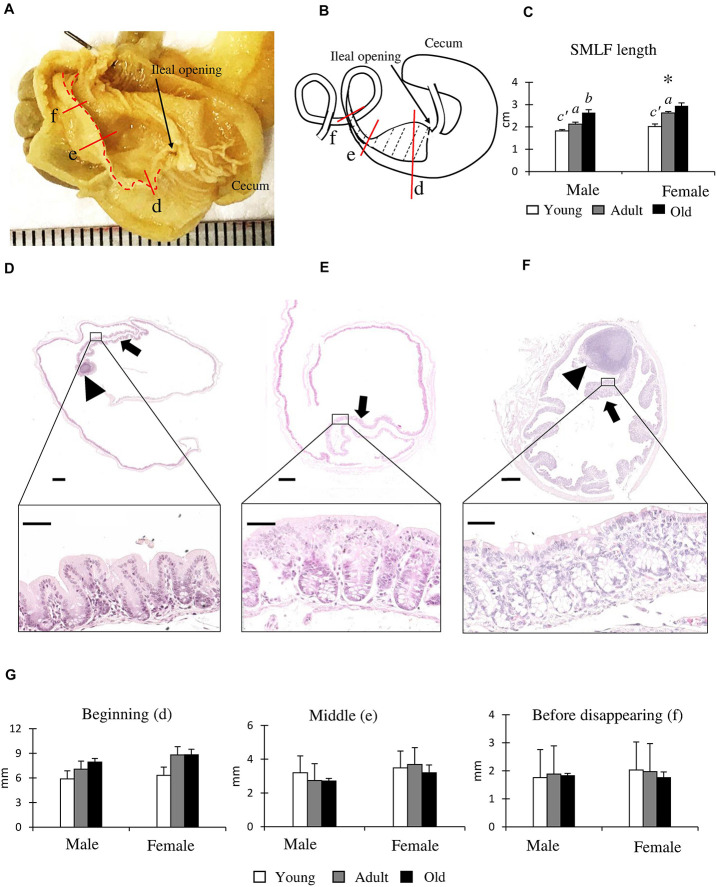
Morphological and histological features of the single longitudinal mucosal fold (SLMF) in the ascending colon (AC) of cotton rats. **(A)** Inner features of the cecum to the beginning of the SLLF. The SLMF is indicated by a dotted line. **(B)** Localization of the SLMF. The SLMF is indicated by a dotted-line area. **(C)** SLMF length. Values are presented as the mean ± standard error.*n* = 21 (male, young age group), *n* = 12 (female, young age group), *n* = 11 (male, adult age group), *n* = 8 (female, adult age group), *n* = 6 (male, old age group), and *n* = 7 (female, old age group). Significant sex-related differences in the same age group are indicated by ^∗^ (*P* < 0.05; Mann-Whitney *U* test). Significant differences between the young and adult groups are indicated by *a* or *a’*. Significant differences between the adult and old groups are indicated by *b* or *b’* (*a, b P* < 0.05; *a’, b’ P* < 0.01; Kruskal-Wallis test followed by Scheffé’s method). **(D–F)** Histological features of SLMFs. Panels **(D–F)** correspond to the areas shown with red lines indicated in Panels **(A,B)**. Beginning of the SLMF just after ileal opening **(D)**, middle position of the SLMF where the colon begins to decrease its diameter **(E)**, 3 mm before disappearing SLMF **(F)**. Arrows and arrowheads indicate the SLMF and lymphatic nodule (LN), respectively. The lower panels indicate the high-magnification area of the square area in the upper panels. Hematoxylin and eosin staining. **(G)** SLMF height. Values are presented as the mean ± standard error.*n* = 4 (male, young age group), *n* = 5 (female, young age group), *n* = 4 (male, adult age group), *n* = 5 (female, adult age group), *n* = 4 (male, old age group), and *n* = 5 (female, old age group).

Figure 4A shows the inner features from the cecum to the AC in adult males (4 months old). The SLMF originated from the ileal papilla to the beginning of the SLLF and was located along the mesenteric border (Figures 4A,B). The length of the SLMF is shown in Figure 4C: it significantly increased with age in both sexes, with females having a significantly longer SLMF than males in the adult group. We examined the histology of the AC with SLMF by dividing it into three parts (Figures 4D–F). The SLMF was lined with the LI epithelium and the number of goblet cells increased from the beginning of the SLMF to the SLLF. Some areas of the SLMF possessed several LNs in their lamina propria (Figures 4D,F). Subsequently, the SLMF height was measured in three different parts of the AC (Figures 4D–G); its height gradually diminished toward the distal part of the AC (Figures 4D–F) in both sexes, and all groups had a decreased luminal diameter of AC. However, there were no sex- or age-related differences.

Additionally, we confirmed the absence of the SLMF in hamsters (cotton rats and hamsters belong to the same family: Cricetidae) and mice (Supplementary Figure S3). Hamsters and mice had a transverse mucosal fold (semilunar shaped) that began near the cecal side of the ileal opening and continued up to the beginning of the AC, which partly separated the cecum and AC. Lastly, we established the appearance of the SLMF in neonatal (0 days old) and 4-day-old cotton rats (Supplementary Figures S3G–I).

**FIGURE 5:**
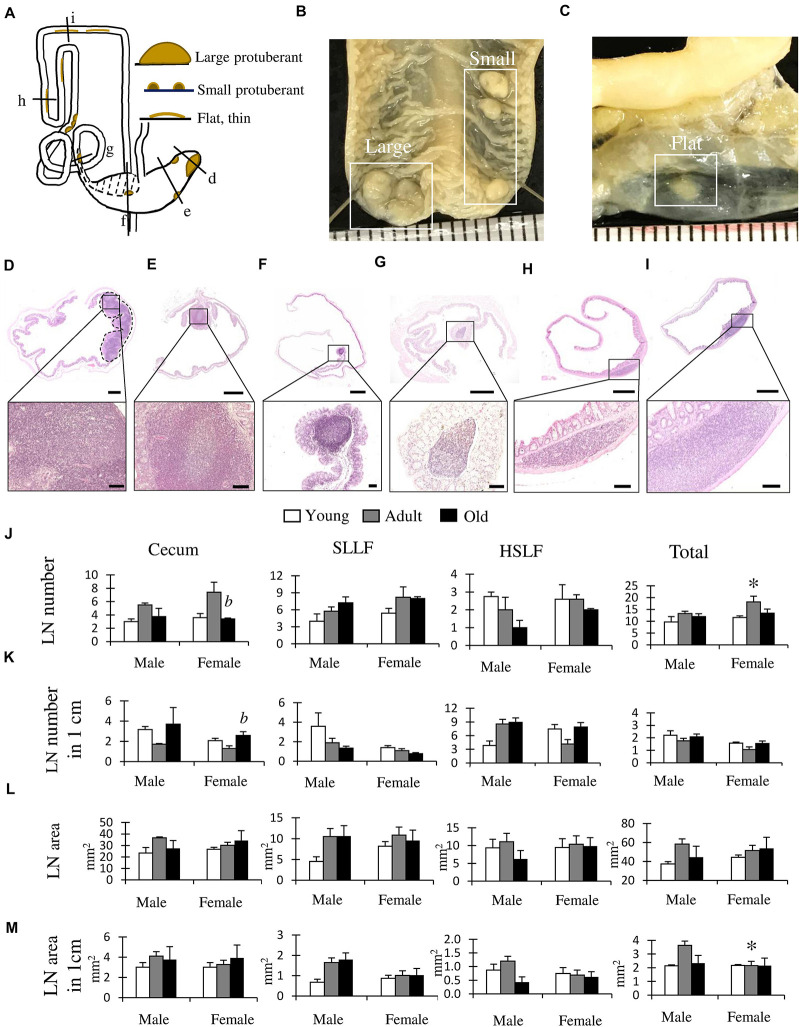
Morphological and histological features of lymphatic nodules (LNs) in the cecum and colon of cotton rats. **(A)** Summarized localization and shapes of LNs found in the cecum and colon. Dotted line area indicates the localization of the single longitudinal mucosal fold (SLMF). Ventral view. **(B,C)** Gross anatomical features of LNs found in the cecum **(B)** or colon **(C)**. Ruler = 1 mm. **(D–I)** Histological features of the LN. Panels **D–I** correspond to the areas indicated with black lines in panel **A**. Near the apex of cecum **(D)**, middle position of cecum **(E)**, after ileal opening **(F)**, before end of spiral loop-like flexure (SLLF) **(G)**, horseshoe-like flexure (HSLF) **(H)**, and transverse colon **(I)**. The lower panels indicate magnified images of the square areas in the upper panels. Hematoxylin and eosin staining **(J–M)** LN number **(J)**, LN number in unit length **(K)**, LN area **(L)**, and LN area in unit length **(M)**. Values are presented as the mean ± standard error*. n* = 4 (each group). Significant sex-related differences in the same age group are indicated by ^∗^*P* < 0.05; Mann-Whitney *U* test. Significant differences between the adult and old groups are indicated by *b* (Kruskal-Wallis test followed by Scheffé’s method).

We focused on the LNs of the LI (Figure 5) and found three types of LNs classified by their size and shape (Figure 5A). First, a large protuberant LN was located near the apex of the cecum (Figures 5A,B; large-type). Second, small protuberant LNs were found from the cecum to the beginning of the HSLF (Figures 5A,B; small-type). Third, thin, flat LNs were observed in the HSLF and transverse colon (Figures 5A,C; flat-type).

Histologically, large-type LNs showed aggregation with germinal centers in the lamina propria to the submucosa at the apex of the cecum (Figure 5D). Small-type LNs were found in the lamina propria from the cecum to the beginning of the HSLF with a germinal center, mainly at the top of the beginning and base of the end part of the longitudinal fold (Figures 5E–G). Flat-type LNs were observed in the lamina propria of the HSLF and transverse colon without a germinal center (Figures 5H,I).

Figures 5J–M shows the results of the morphometric analysis of the number and size of LNs in the LI, including the cecum, SLLF, and HSLF, and their total data. For the LN number, female rats showed a significantly higher number in adults than in old groups in the cecum, and the total number was significantly higher in females than in male adult rats (Figure 5J). Regarding the ratio of LN number to LI unit length, female rats had significantly higher values in the old group than in the adult group in the cecum, indicating a higher density of cecum LNs (Figure 5K). For this parameter, there were no significant sex- or age-related differences. For the LN area or the ratio of LN area to LI unit length, there were no significant age-related differences in each part (Figures 5L,M), however, females had significantly lower values than males in the total values of the ratio of LN area to LI unit length (Figure 5M).

## Discussion

We clarified the morphological features of the LI in cotton rats. Sex differences were found in the AC, and the presence of the SLLF and HSLF was unique to cotton rats compared to other mammals and contributed to increasing the total length of the LI. Similar SLLF and HSLF structures were observed in hamsters but not in mice (Supplementary Figure S1). Similar to other mammals, rodent feeding behavior, digestive tract morphology, and body weight are closely associated (Naya et al., 2014). Laboratory mice originate from the wild *Mus musculus* and are usually omnivorous animals. Syrian hamsters, *Mesocricetus auratus*, are also omnivorous rodents, originating from northwest Syria, especially from dry and stony areas. Their natural diet mainly includes grains, and occasionally insects, fruit, and green plants (Mark et al., 2012). The cotton rat is a member of the Cricetidae family, which includes hamsters. These animals are mainly distributed in the southern United States, Mexico, Central America, Columbia, and Venezuela, in areas that are commonly surrounded by grassy, dense forests, and marshland areas. Cotton rats are omnivorous and their diet principally consists of plants and seeds, and they only occasionally eat invertebrates, eggs, and small birds (Curlee and Cooper, 2012). The food habits of the animals, including laboratory rodents, would be reflected in their LI morphology, especially in the AC, because herbivores LIs develop to digest grass (high-fiber food). The ratio of the LI to the small intestine was larger in the hamster, cotton rats, and mice, in that order (Supplementary Figure S2). These data might therefore reflect omnivory in mice, omnivory with a tendency toward herbivory in hamsters, and intermediate diet type in cotton rats.

At the beginning of the AC, we found an SLMF possessing several protuberant or small-sized LNs; the SLMF grew in length but not in height with the age of the animals. The presence of the SLMF in neonatal cotton rats suggests that the mucosal fold is an innate structure and is not dependent on feeding behavior for its development in this species. In mammals, the colon is part of the LI, which contains the largest population of microorganisms in the GIT, and plays a major role in the absorption of liquid and minerals (Stevens, 1978). Kararli (1995) found that colon anatomy significantly differed among species. Furthermore, numerous rodents were found to have various shaped mucosal folds in the AC that enable the formation of wrinkles, which maintain their nutrient supply system. In general, in comparison to large animals, small herbivorous animals require a special digestive strategy in the LI to utilize plant nitrogen in a short time, owing to their higher metabolic rate, restriction of passage speed, and short digestive transit time (Sakaguchi, 2003). As studied by Sperber (1985) and Sakaguchi (2003), this digestive strategy comprises a colonic separation mechanism that separates colonic contents by digestion rate. This allows coarse particles to be distinguished and eliminated in a short time through the digestive tract, while maintaining the accumulation of microorganisms in the cecum. This mechanism has been established in different species of mammals and birds (Björnhag, 1989), including rabbits (Björnhag, 1981), marsupials (Björnhag, 1994), Scandinavian lemmings, rats (Sperber et al., 1983), guinea pigs, and chinchillas (Holtenius and Björnhag, 1985). Our findings show that the morphological and histological structures of the colonic mucosal fold in several rodent (mouse, cotton rat, hamster) species are similar to those reported in other rodent species, and this is consistent with what is expected in omnivores.

We found various types of LN in the cecum and AC of cotton rats, including protuberant and flat nodules. The lymphoid tissue, including the patch type or solitary intestinal lymphoid tissue (SILT), has been well studied in the small intestine (Buettner and Lochner, 2016). For the murine LI, colonic patches that are developed during the embryonic stage are positioned in the submucosa between the muscular layers and muscular mucosa. SILT, which originates during the postpartum period, is located in the lamina propria, making direct contact with the intestinal epithelium, toward the intraluminal area (Baptista et al., 2013). Therefore, flat LNs in the HSLF and transverse colon and protuberant LNs in the cecum and SLLF might be types of colonic patch and SILT, respectively. These localization patterns indicate the developmental differences of LNs in the LI. The shape of the LNs might also be related to the hardness of feces in the LI. The water content of feces gradually decreases from the beginning of the colon to the rectum; therefore, the physical pressure of feces on the intestinal wall differs among the LI regions. LNs were localized to the SLMF and an appendix was not observed, however, we found large LNs near the apex of the cecum, surrounded by several small LNs. Thus, these data emphasized the crucial role of the cecum and AC in mucosa-associated lymphoid tissue, potentially serving as a “safe house” (Bollinger et al., 2007) or reservoir for commensal bacteria in case of normal microbiome and immunological barrier function loss (the appendix in primates and rabbits).

Adult female cotton rats showed a significantly longer AC with a higher number of SLLFs compared to males. SLMF length was also longer in adult females. However, sex-related differences were not found in mice and hamsters. We assume that sex differences were only identified in cotton rats due to the genetic background, feeding behavior, and geography of the original wild individuals. These results are a critical and valuable finding of the existence of sex-related differences in the LI lining. Sex-related hormones are strongly correlated with gut biology. Androgen and estrogen receptors are expressed in GIT cells, including the interstitial cells of the lamina propria and submucosa (Palsson and Whitehead, 2012) and epithelial cells in the gastric glands, small intestines, and LI (Winborn et al., 1987). Estrogen receptor β (ERβ) is the dominant estrogen receptor and is expressed in the colonic mucosal tissues of humans, especially in vascular smooth muscle and endothelial cells (Konstantinopoulos et al., 2003). In humans, ERβ receptors are known to regulate intestinal barrier function (Looijer-van Langen et al., 2011) and could play an important role in diseases showing sex differences, such as colorectal cancer (where ERβ is more highly expressed in females than in males; Nüssler et al., 2008) and inflammatory bowel disease (Kim and Kim, 2018). In addition, the total excretion of bile acid in humans is lower, the digestive transition time is slower, the ability to digest neutral detergent fiber is higher, and stool consistency is harder in females than in males (Lampe et al., 1993). Here, for the first time, we found sex-related differences in the LI length and its lining in cotton rats. Data is limited for comparing our study to, however, this species could play a crucial role in elucidating the mechanisms behind LI diseases that show sex differences.

In conclusion, cotton rats are one of the most valuable experimental rodents. Cotton rat LIs showed a unique morphological structure and pattern associated with the presence of the SLLF, HSLF, and SLMF, and various types of LNs and sex-related differences. Thus, this rodent may serve as a principal model for elucidating species-specific digestive tract function, gastrointestinal disorders, and their sex-related characteristics. Further studies are required to investigate the mechanisms of sex differences in the AC.

## Data Availability Statement

All datasets presented in this study are included in the article/ Supplementary Material.

## Ethics Statement

The animal study was reviewed and approved by Hokkaido Institute of Public Health.

## Author Contributions

YK and OI supervised the findings of this study. TI supplied the samples of cotton rats. YO-O supplied the hamster samples. YE, TeN, TaN, MI, MM, and YO provided the critical feedback and helped shape the research, analysis, and manuscript. TC, OI, and YK analyzed the data and wrote the manuscript. All authors contributed to the article and approved the submitted version.

## Conflict of Interest

The authors declare that the research was conducted in the absence of any commercial or financial relationships that could be construed as a potential conflict of interest.

## Abbreviations

AC: ascending colon
GIT: gastrointestinal tract
HSLF: horseshoe-like flexure
LI: large intestine
LN: lymphatic nodule
SILT: solitary intestinal lymphoid tissue
SLLF: spiral loop-like flexure
SLMF: single longitudinal mucosal fold.
