# Supplementary material for: Unique Running Pattern and Mucosal Morphology Found in the Colon of Cotton Rats
Source: Front Physiol. 2020 Oct 26;11:587214. doi: 10.3389/fphys.2020.587214 (PMC7649294; doi:10.3389/fphys.2020.587214)
Supplement: Supplementary file 1 [file Image_1.pdf]

# *Supplementary Material*

**Supplemental Figure 1. Morphology of large intestines in different rodents.**

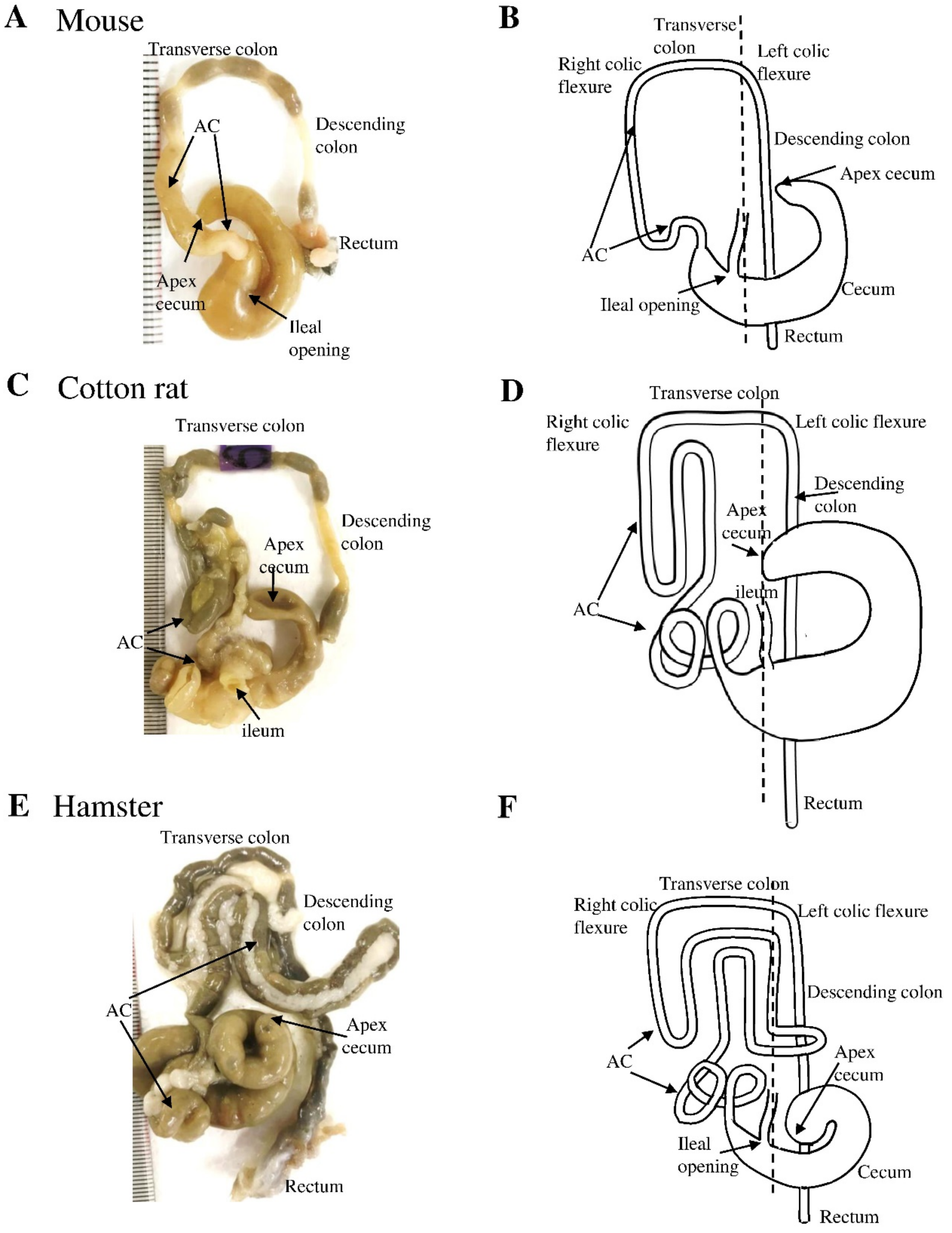

(**A and B**) Male mouse at 2 months. Panel (A) shows a picture from the ventral view. Panel (B) summarizes the morphology and running pattern of the large intestine from the ventral view. (**C and D**) Male cotton rat at 6 months. Panel (C) shows a picture from the ventral view. Panel (D) summarizes the morphology and running pattern of the large intestine from the ventral view. (**E and F**) Male hamster at 6 months. Panel (E) shows a picture from the ventral view. Panel (F) summarizes the morphology and running pattern of the large intestine from the ventral view. AC: ascending colon. Ruler = 1 mm.

**Supplemental Figure 2. Length of small and large intestines in different rodents.**

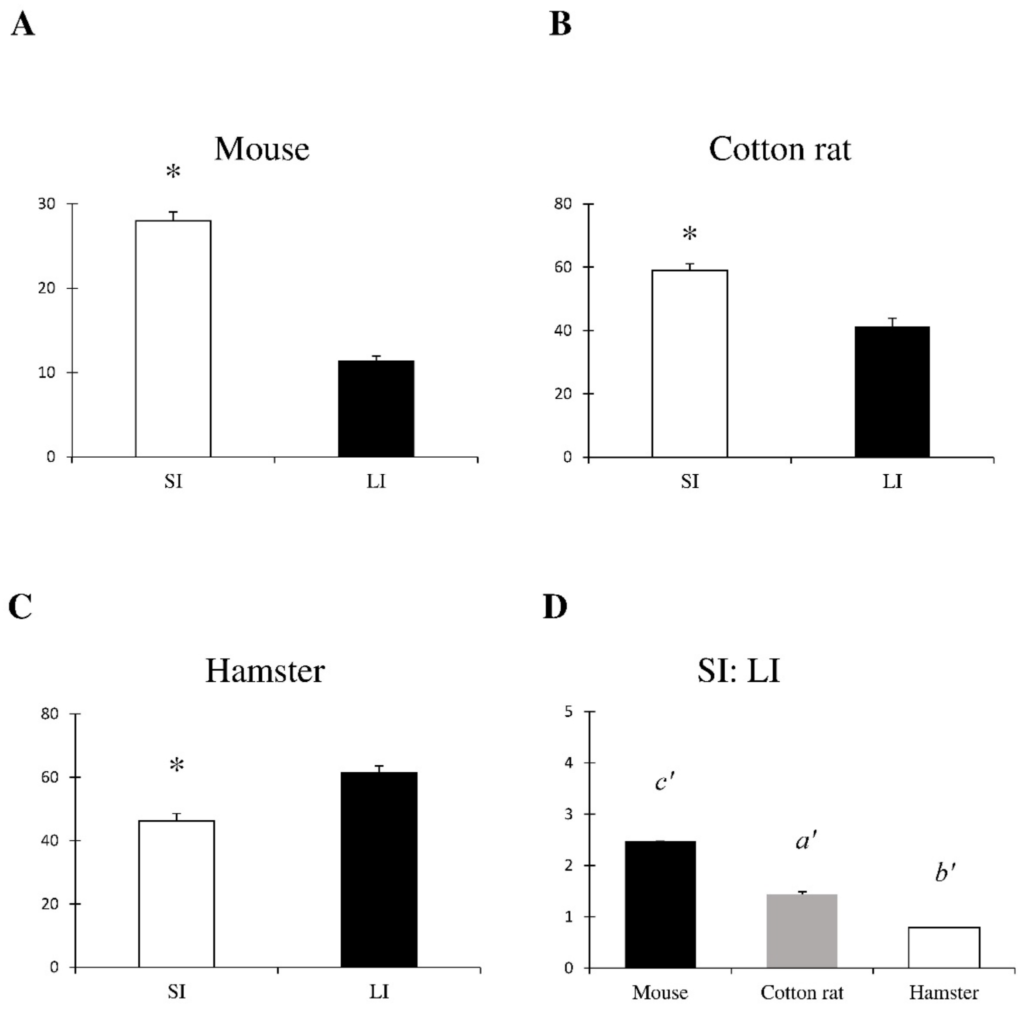


(**A**) Mouse at 2 months. (**B**) Cotton rat at 6 months. (**C**) Hamster at 6 months. (**D**) Ratio of small intestine to large intestine (LI) length. Values are presented as mean ± standard error. $n = 4$ (each group). Significant differences between the small intestine and LI are indicated by * ($P < 0.05$) (Mann-Whitney *U* test). Significant differences between the young and adult groups are indicated by *a*. Significant differences between the adult and old groups are indicated by *b.* Significant differences between the old and young groups are indicated by *c.* ($P < 0.05$) (Kruskal-Wallis test followed by the Scheffé's method).

**Supplemental Figure 3. Mucosal folds found in rodent colons.**

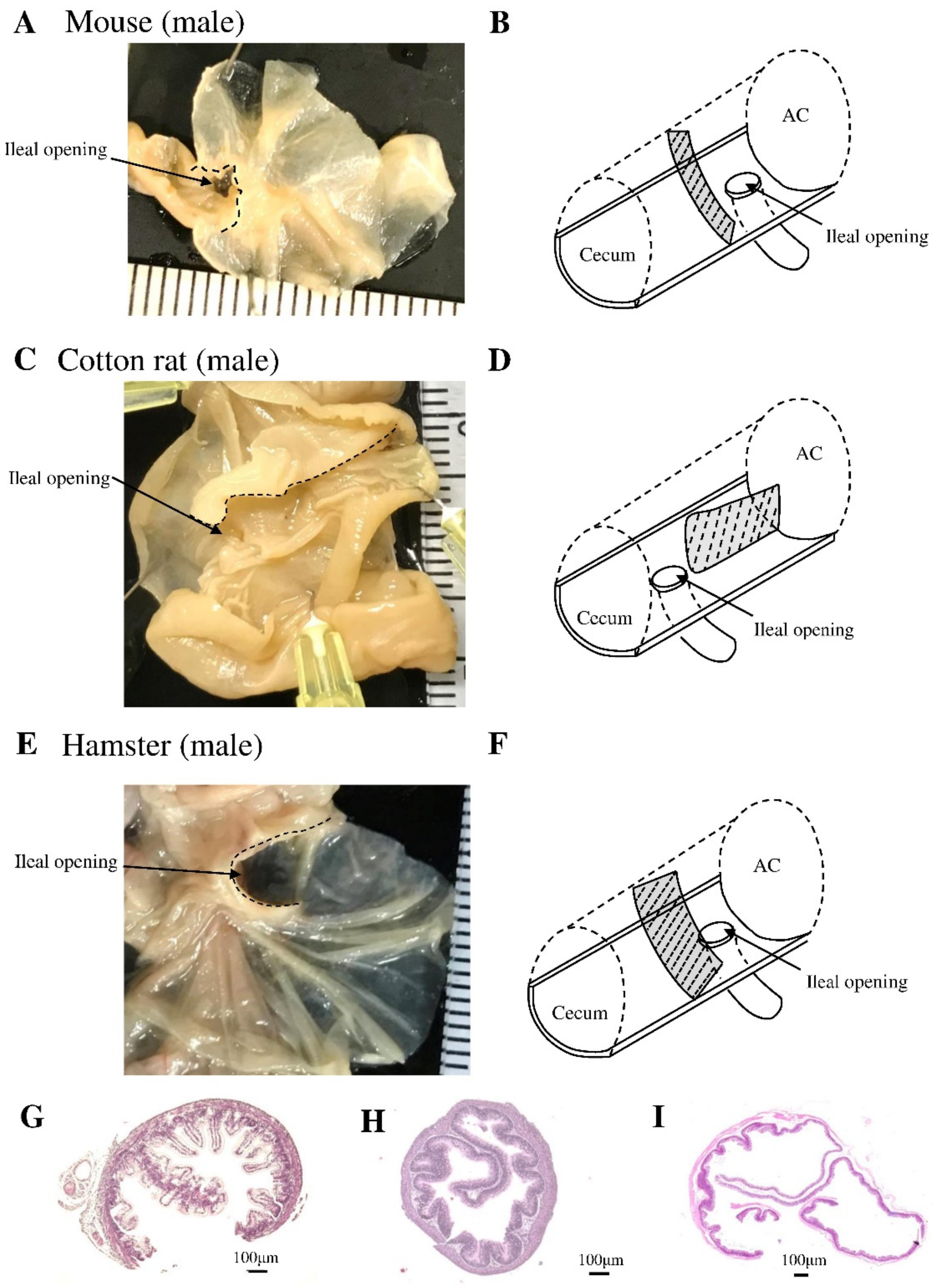


(A and B) Male mouse at 2 months. Panel (A) shows a picture of the inner features at the border between the cecum and colon. Panel (B) summarizes the morphology at the border between the cecum and colon. The gray dotted line area indicates the simple mucosal fold. (C and D) Male cotton rat at 6 months. Panel (C) shows a picture of the inner feature at the border between the cecum and colon. Panel (D) summarizes the morphology at the border between the cecum and colon. The gray dotted line area indicates the simple mucosal fold. (E and F) Male hamster at 6 months. Panel (E) shows an image of the inner feature at the border between the cecum and colon. Panel (F)

summarizes the morphology at the border between the cecum and colon. The gray dotted line area indicates the simple mucosal fold. (G–I) Single mucosal longitudinal fold of cotton rats at 0 day (G), 4 days (H), and 4 months (I). Hematoxylin and eosin staining.
